# Evaluation of dermatological asymmetry measure of shape by histogram energy measure

**DOI:** 10.3389/fimmu.2026.1768346

**Published:** 2026-09-08

**Authors:** Łukasz Wąs, Piotr Milczarski

**Affiliations:** 1 Institute of Mechatronics and Information Systems, Lodz University of Technology, Lodz, Poland; 2 Institute of Information Technology, Lodz University of Technology, Lodz, Poland; 3 Faculty of Mathematics and Computer Science, University of Lodz, Lodz, Poland

**Keywords:** dermatological asymmetry measure, dermatological features segmentation, skin lesions classification, skin lesions clusterization, symmetry and asymmetry of skin lesion

## Abstract

**Introduction:**

This study addresses automatic detection of skin changes especially asymmetry and symmetry of the lesions and their crucial role in the definition of dermatological asymmetry measure.

**Method:**

The present research employs an integrated methodology, combining systematic evaluation of skin lesions. In the paper, we use unsupervised learning methods to assess the asymmetry of the lesions for dermatology as tool for computer-aided diagnostic systems. The asymmetry features are extracted from the images as vectors and then calculated using two dermatological shape asymmetry measure (DASMShape) methods. In the next step, to evaluate the clusters and their clusters to classes assignment, we have used classification methods: Support Vector Machines (SVM), k-nearest neighbors (kNN), Random Forrest, Multilayer Perceptron, and C4.5 to assess defined above dermatological asymmetry procedure.

**Results:**

This multi-analytical approach generates comprehensive data on skin lesion analysis. The proposed approach effectively combines shape asymmetry (DASMShape) with texture analysis (Gabor filter energy) and intensity distribution to create a comprehensive diagnostic profile The high classification results --reaching up to 100% in certain configurations --confirm that unsupervised learning can accurately group lesions into diagnostic categories that align with expert.

**Discussion:**

This system represents a significant step toward bridging the gap between general practitioners and specialists, providing a tool that prioritizes high sensitivity while maintaining the analytical depth required to reduce false negatives in melanoma screening.

## 1 Introduction

The identification and early diagnosis of skin changes, particularly malignant melanoma and non-melanoma skin diseases, represent a critical challenge in contemporary dermatology (1, 2). Because early detection is a fundamental factor in improving patient survival rates, developing reliable screening methods is a primary objective for medical researchers (3). According to data from the European Cancer Information System (ECIS) (2), the estimated risk of melanoma in 2018 varied significantly across the continent, with rates as high as 38.2 new cases per 100,000 population in Germany (1). Similarly, the American Cancer Society (ACS) reported that the lifetime risk of developing melanoma is approximately 1 in 27 for males and 1 in 42 for females (1).

In clinical practice, dermatologists utilize several established screening protocols to evaluate suspicious lesions, including the ABCD rule, the Seven-Point Checklist (7PCL), the Three-Point Checklist of Dermoscopy (3PCLD) (3–5), the ABCD rule, CASH, and so forth (6). These methods rely on the identification of specific dermoscopic features such as pigment networks, dots, globules, and streaks, as well as the assessment of asymmetry in shape, color, and structure (2, 4). In particular, the 3PCLD is designed as a simplified screening technique for non-experts, focusing on three criteria: asymmetry in shape, hue, and structure; atypical pigment network; and blue-white structures. While this method achieves a high sensitivity rate of 96.3% even when used by non-experts, the true negative rate for non-experts remains significantly lower (32.8%) compared to that of specialists (94.2%) (4).

This disparity in diagnostic accuracy suggests that general practitioners and non-experts require robust support systems (4). Automated computer-aided diagnostic (CAD) systems have emerged as a vital tool to assist in the early detection of suspicious lesions (4, 7, 8). However, the computational measurement of symmetry—a core component of these diagnostic rules—is complex. While axial symmetry was historically the first approach used in computer science for shape description, modern methods have evolved to include local contour curvature, topographical analysis, and Gaussian derivatives to determine global symmetry (9). In the context of dermatology, symmetry is not limited to shape alone; it must also account for the distribution of pigmentation and internal structures (5, 6).

The primary objective of this research is to present an automated approach for detecting skin changes using unsupervised learning methods to assess lesion asymmetry (7, 10). This study introduces a procedure where asymmetry features are extracted as vectors and evaluated using two Dermatological Asymmetry Measure of Shape (DASMShape) methods (7, 11). The research utilizes clusterization techniques—specifically Canopy, k-Means (KM), and Expectation-Maximization (EM)—to identify mean values and centroids for which asymmetry measures are calculated (7, 12, 13). To validate these clusters and their assignment to diagnostic classes, the study employs various classification algorithms, including Support Vector Machines (SVM), k-Nearest Neighbors (kNN), Random Forest, Multilayer Perceptron, and C4.5 (7, 12, 14). Nowadays, there are several new approaches to melanoma detection described by Jain (11), Ra (12), Dalila (13), Vincent (14), and Ramlakhan (15).

In the conducted analysis, unsupervised learning methods to assess the asymmetry of the lesions are used. The asymmetry features are extracted from the images as vectors and then calculated. Two DASMShape methods are applied. The clusterization methods usually provide us with some mean values or center points of the centroids. For those points, the asymmetry measures are calculated according to Wąs et al. (9) and Milczarski et al. (8, 16–19). In the next step, to evaluate the clusters and their clusters-to-classes assignment, classification methods: SVM, kNN, Random Forest, Multilayer Perceptron, and C4.5 are used to assess the defined above dermatological asymmetry procedure. In the research, several clusterization methods were tested. Finally, three methods were selected: Canopy, k-Means (KM), and Expectation-Maximization (EM) (20).

The paper is organized as follows. In Section 2, a general approach to skin lesion recognition and classification is presented. Section 3 shows the dermoscopic asymmetry measure as a function of shape, hue/color, and structure. In addition, the results of the clusterization and its assessment by the different asymmetry of shape functions are shown in Section 4. Conclusions are also drawn in that final section.

## 2 Symmetry classification methods

The first works of symmetry in the computer science area appeared with the development of the computers in 40’s and 50’s of the 20th century. The symmetry axial transform is considered the first intuitive choice of the method for the detection of symmetry (21), but there are many other approaches. We can use the local contour curvature to determine global symmetry exploiting the locus of mid-edge-point pairs (22). Symmetry lines can be detected by a topographical analysis of the vector potential field (23). Another method is a calculation of the symmetry measure at each point using the first and second derivatives of Gaussians (24). A symmetry descriptor of an object can be based on a cross-correlation operator evaluated on the gray levels. The authors also use the scale-invariant feature transform (25), complex moments based on the Fourier or Gabor transforms of the images (26). Minimizing the asymmetric term of the energy over an image (27) or “symmetry kernel” (28) is used. The examples of the methods of symmetry applied for the images can be based on textures’ rotation invariance (16).

For melanoma asymmetry, there are several approaches, like in Hernandez (29) and Milczarski (17, 19). The skin lesion asymmetry measure methods can be divided into two groups. The first approach is based on a description between several regions in reference to a set of axes, for example, in (30, 31). The second approach uses region convexity from which asymmetry is inferred (32). Asymmetry can be measured by applying a minimal boundary box on the automatically extracted skin lesion as in (33).

Dermatologists use several diagnostic procedures for disease symptom recognition. The 3-point checklist, the 7-point checklist, and the ABCD rule are used. Regardless of the method, symmetry of the shape of the skin lesion is insufficient to decide about the lesion character. It is necessary to consider the symmetry of the skin lesion, which consists of shape symmetry and pigmentation symmetry (34).

The axial symmetry of pigmentation can refer to pattern symmetry around any axis through the center of the lesion, like in (35). In the case of skin lesion pigmentation symmetry, the distribution of hue/color and structure around any axis through the center of the lesion or two perpendicular axes is used (5, 35). Different methods bisect the lesion by two lines that are placed 90° to each other. The first line attempts to bisect the lesion at the division of most symmetry, and the other one is placed 90° to it (36).

## 3 Dermatological asymmetry measure of shape, DASMShape

In the papers (16, 17), the following definitions and abbreviations were defined:

- DAS – Dermatological Asymmetry – asymmetry of the shape, hue and structure. In dermatology, as we mentioned above, the value of the asymmetry can be equal to 0 for fully symmetric shapes; 1 for symmetric ones in one axis, or 2 for asymmetric ones.
- DASM – Dermatological Asymmetry Measure – real asymmetry measure depending on the shape, hue/color, and structure.
- DASMShape – Dermatological Asymmetry Measure of Shape symmetry/asymmetry.
- DASMHue – Dermatological Asymmetry Measure of Hue/Color symmetry/asymmetry distribution.
- DASMStruct – Dermatological Asymmetry Measure of Structure symmetry/asymmetry distribution.
- GSSPT – a geometrical shape symmetry precision threshold of the binary mask of the lesion is a threshold that, after the axial transformation, the original binary mask and mirror are the same in at least the threshold value. PSSPT values are real values from<0,1>, for example, 0.95 threshold means that the mirrored images are in 95% cover each other. The bigger the threshold the bigger similarity between mirrored images. The symmetry axis, SAx, depends on the GSSPT as well as the number of the symmetry axes for a given shape.
- NSA – number of symmetry axes depending on GSSPT.
- VoSS – a vector of shape symmetry, it as a vector which coefficients are equal to the number of symmetry axes.

**Figure 1:**
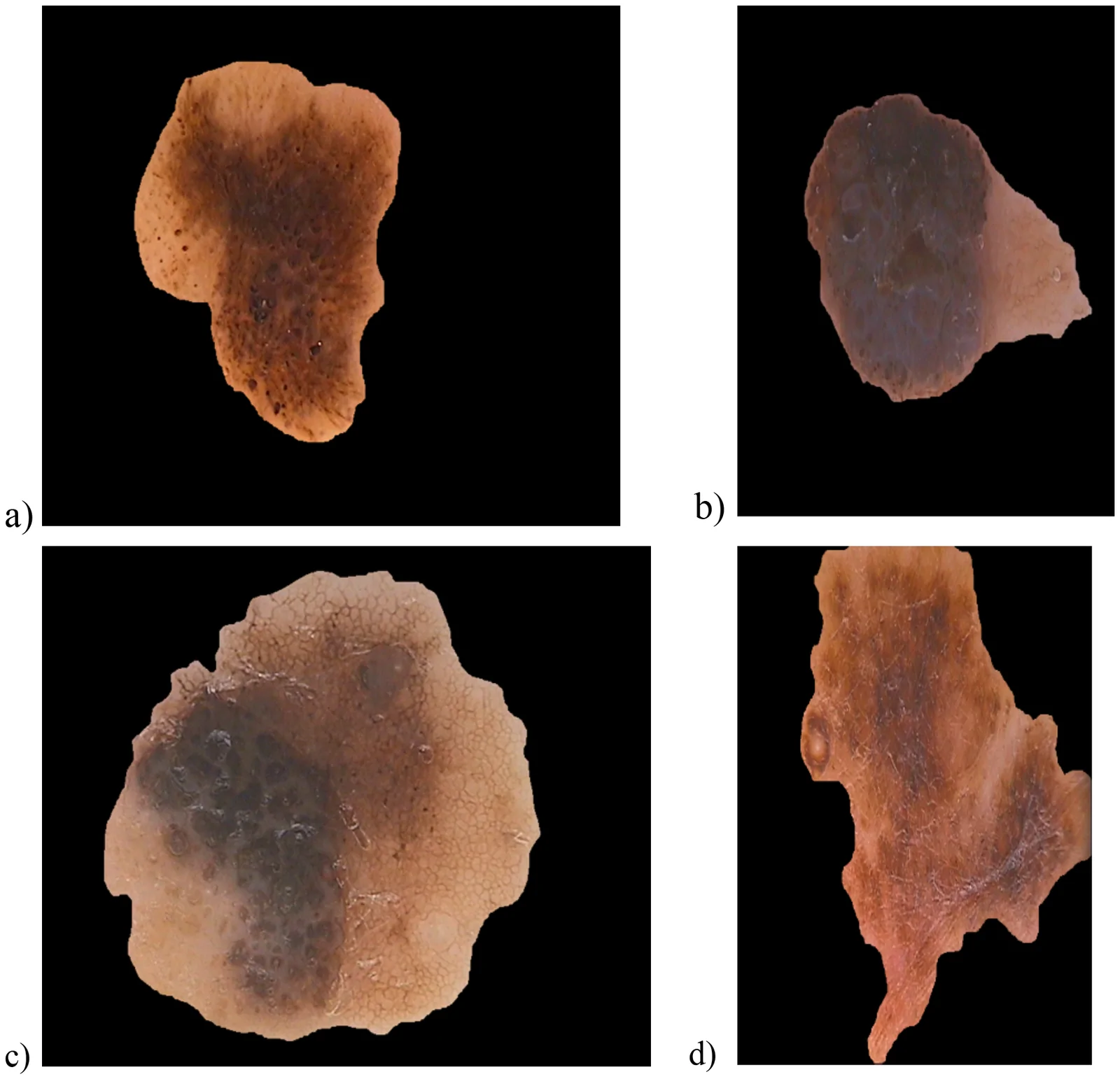
The lesions from PH2 (37) dataset with dermatological asymmetry DAS: **(A)** IMD075 (Atypical nevus) DAS = 2; **(B)** IMD211 (Nodular Melanoma) DAS = 1; **(C)** IMD406 (Nodular Melanoma) DAS = 2; **(D)** IMD404 (Lentigo Maligna) DAS = 2.

**Figures 1A–D** show different binary masks for lesions with their corresponding dermatological asymmetry values assessed by the dermatology experts.

The method presented below was derived by Milczarski in (7). The method of deriving and estimating the new dermatological asymmetry measure value can be described as follows:

1. Calculate the number of symmetry axes for a given set of GSSPT thresholds *n(t_i_)*, where *t_i_* is a given threshold. After our experimental research, we propose to choose the threshold values as a subset of a set of:

*{0.9, 0.91, 0.92, 0.93, 0.94, 0.95, 0.96, 0.97, 0.98}.*

2. From the values of *n(t_i_)* construct a vector of shape symmetry (VoSS) **W**:

(1) $$W=\left[n\left(t_{1}\right),n\left(t_{2}\right),\ldots n\left(t_{k}\right)\right],$$

where *k* ≥ 2.

3. Design the DASMShape, Dermatological Asymmetry Measure of Shape, as a function of VoSS. Because the values of DASM and DASMShape are real values from<0,2>, we propose two types of functions that have positive, normalized to the maximum value of 2, and are continuous measures.

a. The first type is defined as an exponent of function depending on a VoSS vector **W**:

(2) DASMShape(W)=2exp(−f(W)),

where function *f: R^k^* ➔ *R*^+^ ∪ {0}.

b. The second type is defined as rational function depending on a VoSS vector **W**:

(3) $$DASMShape(W)=\frac{2}{f(W)}$$

where inner function *f: R^k^ ➔<1, ∞*).

For each of the DASMShape functions let us introduce a set of two crisp shape thresholds (ST): ST={*lst*, *ust*}, where

(4) $$ST(DASMShape(W))=\begin{array}{c} 0\Leftrightarrow DASMShape(W)<lst\\ 1\Leftrightarrow lst\leq DASMShape(W)<ust\\ 2\Leftrightarrow DASMShape(W)>ust \end{array}$$

The values of *lst* and *ust* will depend on the DASMShape function type and will be derived after optimization of results. The number of misclassified images should have a minimum value parallelly with the number of underestimated cases.

In our experimental research, we have tested several versions of function *f (***W***)* in Equations 2, 3 with different coefficients and for a different subset of GSSPT thresholds for Equation 4. The smaller the number of thresholds values the faster deriving of VoSS vector **W** as defined in Equation 1. We have achieved the best results for the following subset of threshold values:

(5) {0.9,0.93,0.94,0.95,0.97}.

It has to be considered that the bigger the threshold in Equation 5, the more accurate the shape should be acquired to achieve the best asymmetry value.

The tests were conducted using a proprietary java software on a computing station equipped with the processor Intel^®^ Core™ i7-4900MQ CPU @ 2.80GHz, 2801 MHz, with four cores and 8logical processors. The images from the PH2 dataset have 768 × 560 resolution as well as the binary masks used to determine the symmetry/asymmetry axes. The average time to derive the DASMShape of the lesion and their possible axes varied from 0.8 sec to 2.03 sec.

## 4 Exponential type of DASMShape

In the exponential case of the DASMShape, to define the inner function *f(W)* is proposed as follows:

(6) $$f(W)=\sum_{i=1}^{5}a_{i}n_{i}^{2},$$

where values *n(t)* defined for (4) are *n_1_* = *n(t_1_)* = *n(*0.9*),…, n_5_* = *n(t_5_)* = *n(*0.95*).*

In the research, we have tested several vectors of coefficients **a:**

$\text{a}=\left[a_{1},\ldots ,a_{5}\right]$.

We present three examples of them, that are labeled as **a_x_, a_y,_ a_z_** with coefficient shown below

(7) $$a_{x}=\left[0.01,0.025,1/15,1/3,1.0\right]$$

(8) $$a_{y}=\left[0.01,0.02,0.04,0.2,2.0\right]$$

(9) $$a_{z}=\left[0.004,0.01,1/6,1/3,0.5\right]$$

## 5 Rational type of DASMShape

In the rational case of the DASMShape, to define the inner function *f (***W***)* is proposed as follows:

(10) $$f(W)=1+\sum_{i=5}^{5}a_{i}n_{i}$$

where values *n(t)* defined for (4) are *n_1_* = *n(t_1_)* = *n(*0.9*),…, n_5_* = *n(t_5_)* = *n(*0.95*).*

For both DASMShape measures, the values of *lst* and *ust* were researched independently, because they depend on the DASMShape. The examples of Shape Symmetry Vectors **W** for the chosen images from PH2 dataset for different measure functions are provided in **Table 1**. The values in **Table 1** are calculated according to vectors from Equations 7–9, 11, 12 with f(W) calculated by Equations 6, 10. Considering the *f(W)* function type and optimization of the results using the values are:

- Exponential case - *lst* and *ust*
- Rational case - *lst* and *ust*

**Table 1:** Examples of shape symmetry vectors W for images from Ph2 (37) dataset for different measure functions.

Image ID from PH2	VoSS vector W coefficient values					DAS (PH2)	DASMShape values for f(W) and coefficients a				
	n (0.9)	n (0.93)	n (0.94)	n (0.95)	n (0.97)		a_x_	a_y_	a_z_	a_k_	a_m_
IMD075	1	1	1	0	0	**2**	1.80666	1.86479	1.66943	1.78412	1.25000
IMD211	2	1	1	1	0	**1**	1.25627	1.48164	1.18193	1.31406	0.90909
IMD406	13	6	5	2	0	**2**	0.00747	0.02969	0.00290	0.61862	0.28571
IMD404	0	0	0	0	0	**2**	0.0	0.0	0.0	0.0	0.0

In the current paper, unsupervised methods, that is, clusterization are focused on (20).

In **Table 2**, there are classification results of the asymmetry assessment using the following clusterization methods with nine clusters, as it was explained in Sec. III:

**Table 2:** The assessment of the clusterization methods by classification methods, where the best results for k-means (KM) are in bold.

Classifier	Classification results [%]		
	Canopy	KM	EM
3NN	98,7	**98.0**	87.5
C4.5	98.0	**99.3**	98.7
MLP	92.1	**97.4**	89.5
RF	100	**100**	100
SVM	96.1	**96.7**	88.8

- Canopy: max-candidates = 100; periodic-pruning = 10000; min-density = 2.0; T2 radius = 0.804 and T1 radius = 1.005.
- k-Means (KM) with Euclidean distance, max-candidates = 100, periodic-pruning = 10000, min-density = 2.0, T1 = −1.25 and T2 = -1.0.
- Expectation–Maximization (EM) with max-candidates = 100, “minimum improvement in log likelihood” = 1E-5, “minimum improvement in cross-validated log likelihood” = 1E-6, and “minimum allowable standard deviation” = 1E-6.

To validate and to choose the clusterization method, we have used five machine learning methods (12, 13). All the clusterization results were assessed by the classification methods with the same parameters. In **Table 2**, there are classification results of the production processes using the following classifiers:

- 3NN (kNN) 3-Nearest Neighbors.
- Multilayer Perceptron (MLP) with a hidden layer with 16 nodes for the onion and 15 nodes for the spinach production with a learning rate equal to 0.79 and momentum equal to 0.39 (14);
- binary tree C4.5 with a confidence factor equal to 0.25, with a minimum number of instances per leaf equal to 2.
- Random Forest (RF) with the bag size percent equal to 100, with maximum depth unlimited, number of execution slots equal to 1, and 100 iterations.
- Support Vector Machine (SVM) with a radial basis function (RBF) given by the Equation 11:

(11) $$K\left(x,y\right)=\exp\left(-0:05*{\left(\text{x}-\text{y}\right)}^{2}\right)$$

The best clusterization method is k-means, followed by Canopy, see **Table 2**.

## 6 Discussion of the results

**Figure 2:**
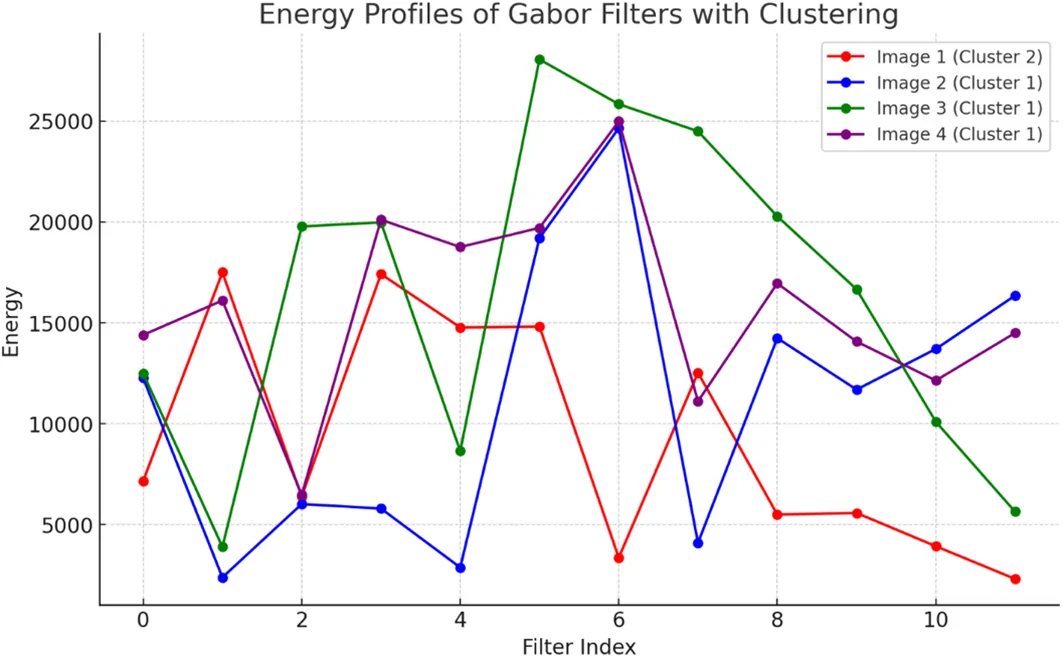
The energy profiles of Gabor filters with clustering.

Let us discuss the energy profiles of Gabor filters with clustering shown in **Figure 2**.

Analysis of the results and their interpretation:

1. Euclidean distances between images (distance matrix):

The distances indicate how much the energy profiles differ between the images:

- The greatest distance is between the First Image (Image 1) and the Latest Image (Image 3) (~40,637).
- The smallest distance is between the Second Image (Image 2) and the Last Image (Image 4) (~26,808).

This confirms that images have unique texture features.

2. Cluster assignments:

The images have been divided into two clusters:

- Cluster 1: Obrazy 2, 3, 4 (Image 2, Image 3, Image 4).
- Cluster 2: Obraz 1 (Image 1).

Image 1 forms a separate cluster, which means that its texture features are significantly different from the others.

3. Key images in cluster centers:

Cluster 1 (Hub): Best represented by Image 4 (latest image).
Cluster 2 (Center): The only representative is Image 1 (first image).

Visualization of energy profiles:

The graph (**Figure 2**) shows the response energy profiles of the Gabor filters for all images.

**Figure 3:**
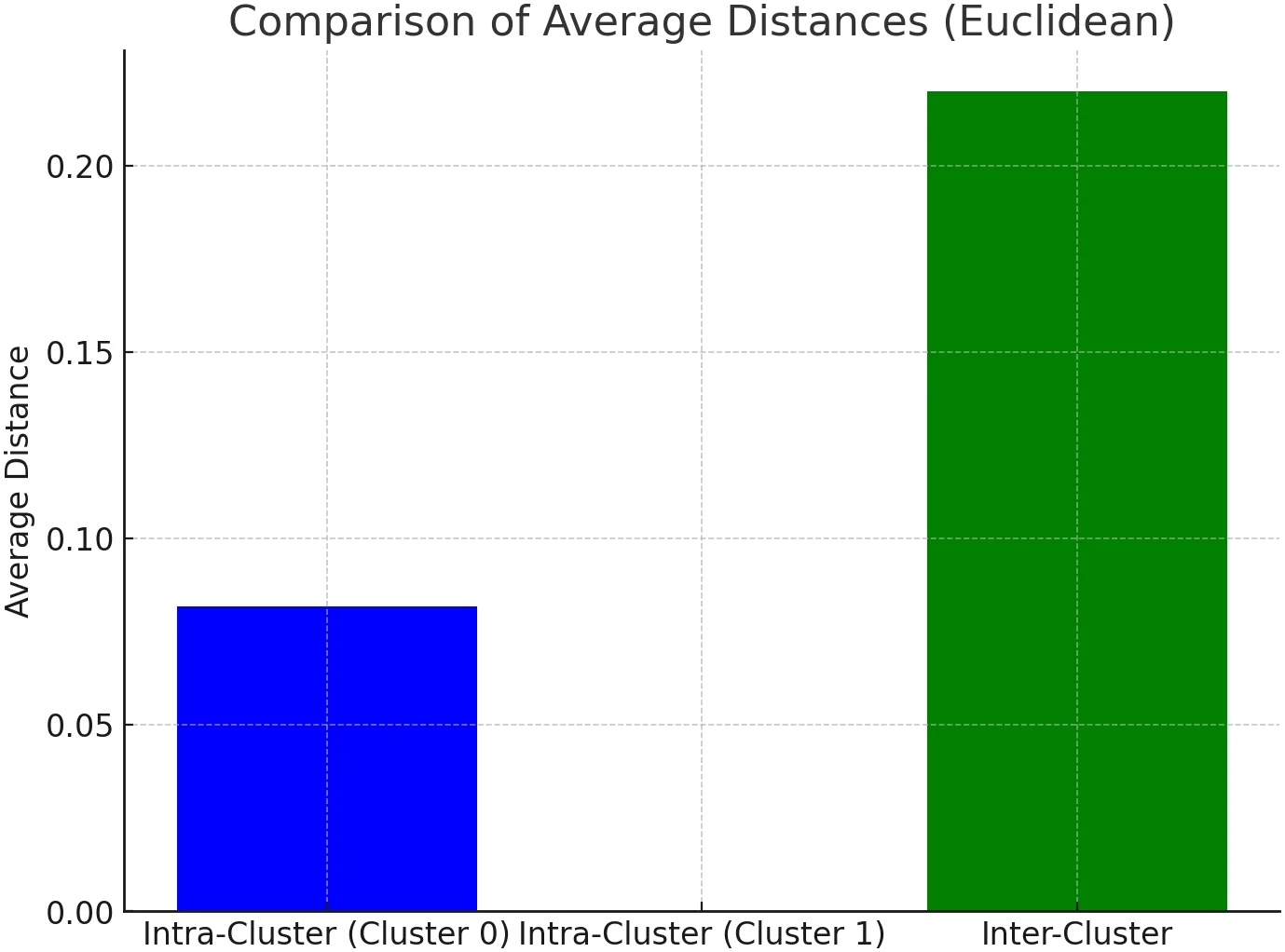
The comparison of the average distances in Euclidean space.

**Figure 4:**
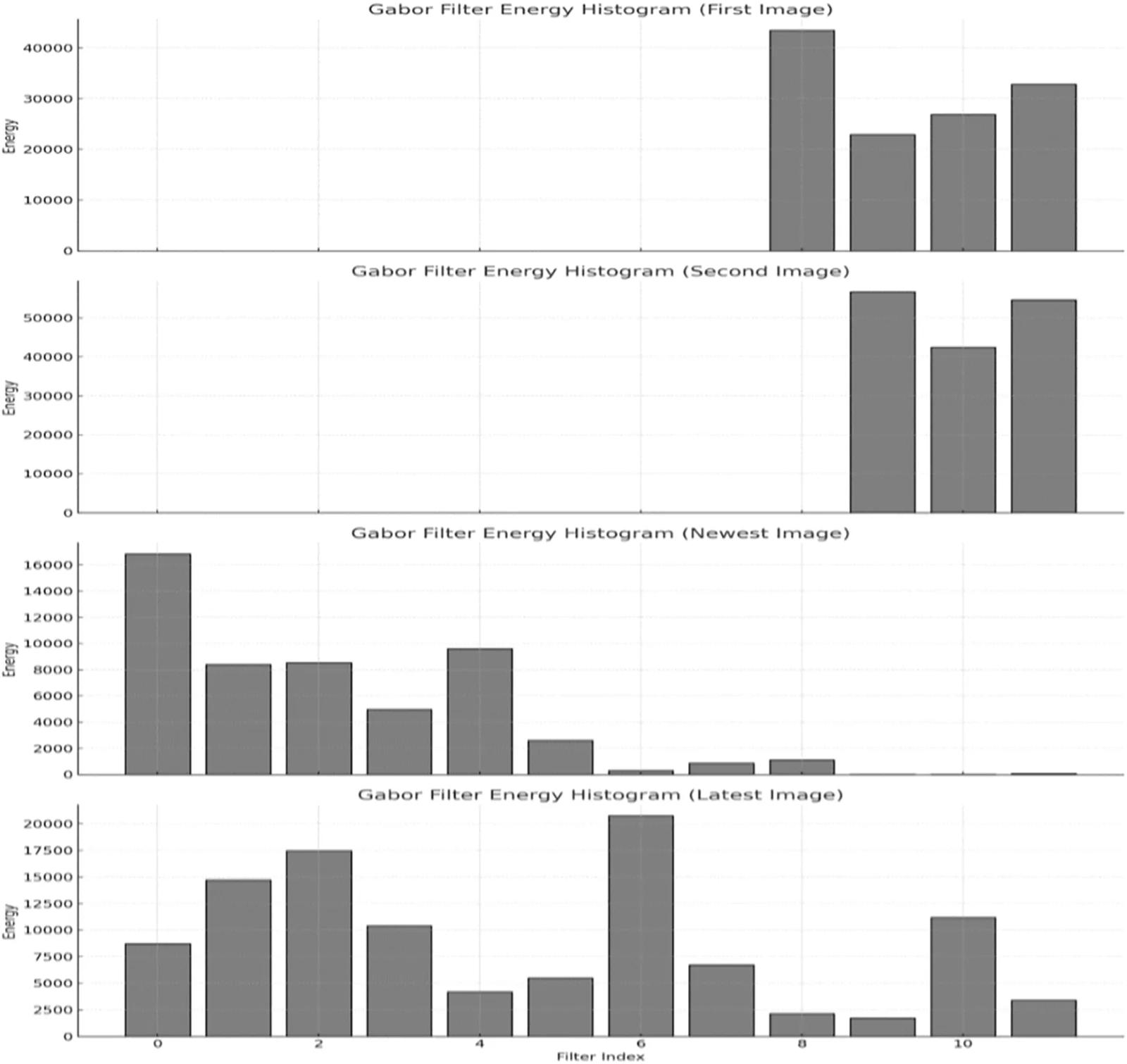
The Gabor filter response energy histograms.

**Figure 5:**
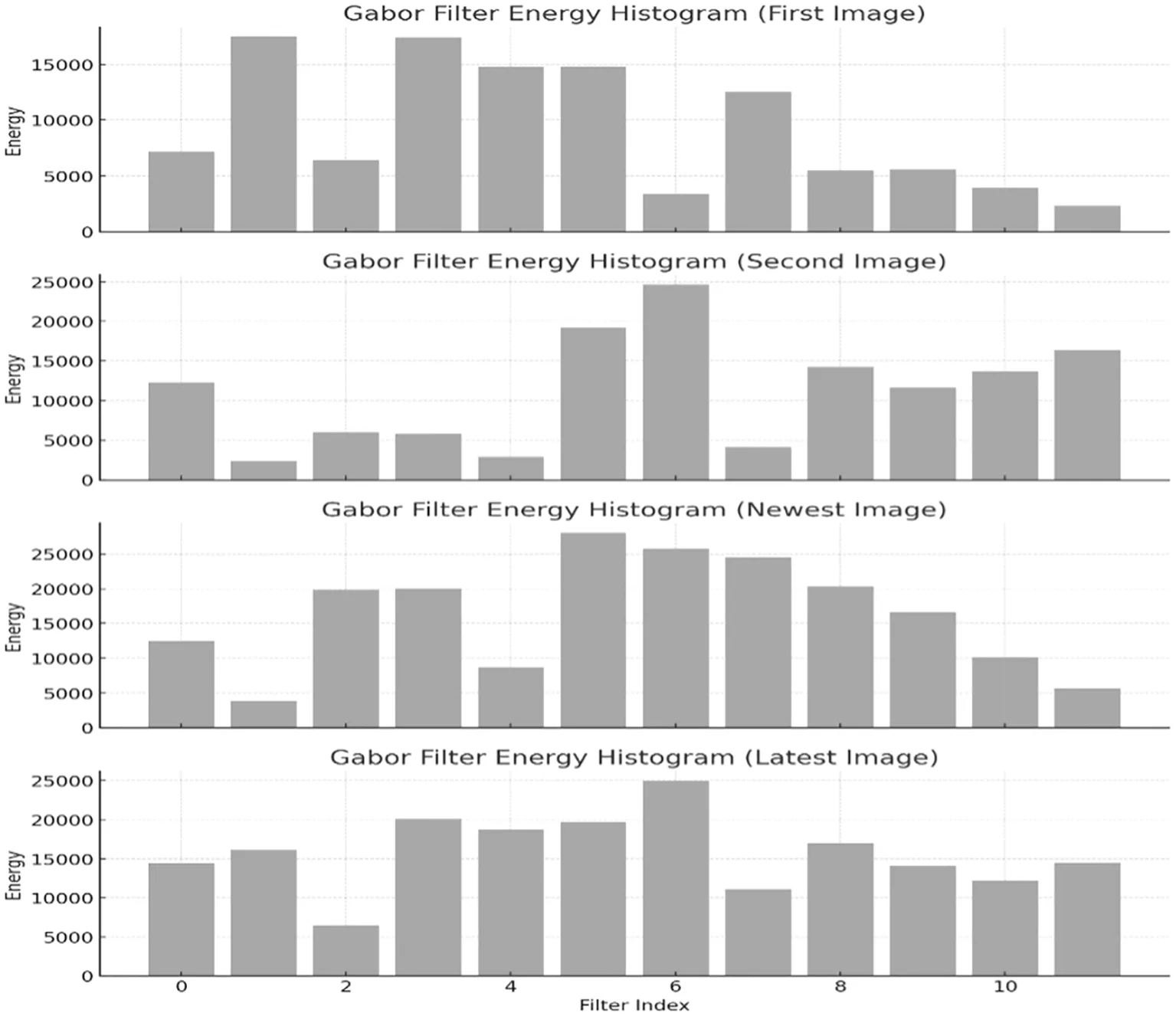
The Gabor filter response energy histograms.

**Figure 6:**
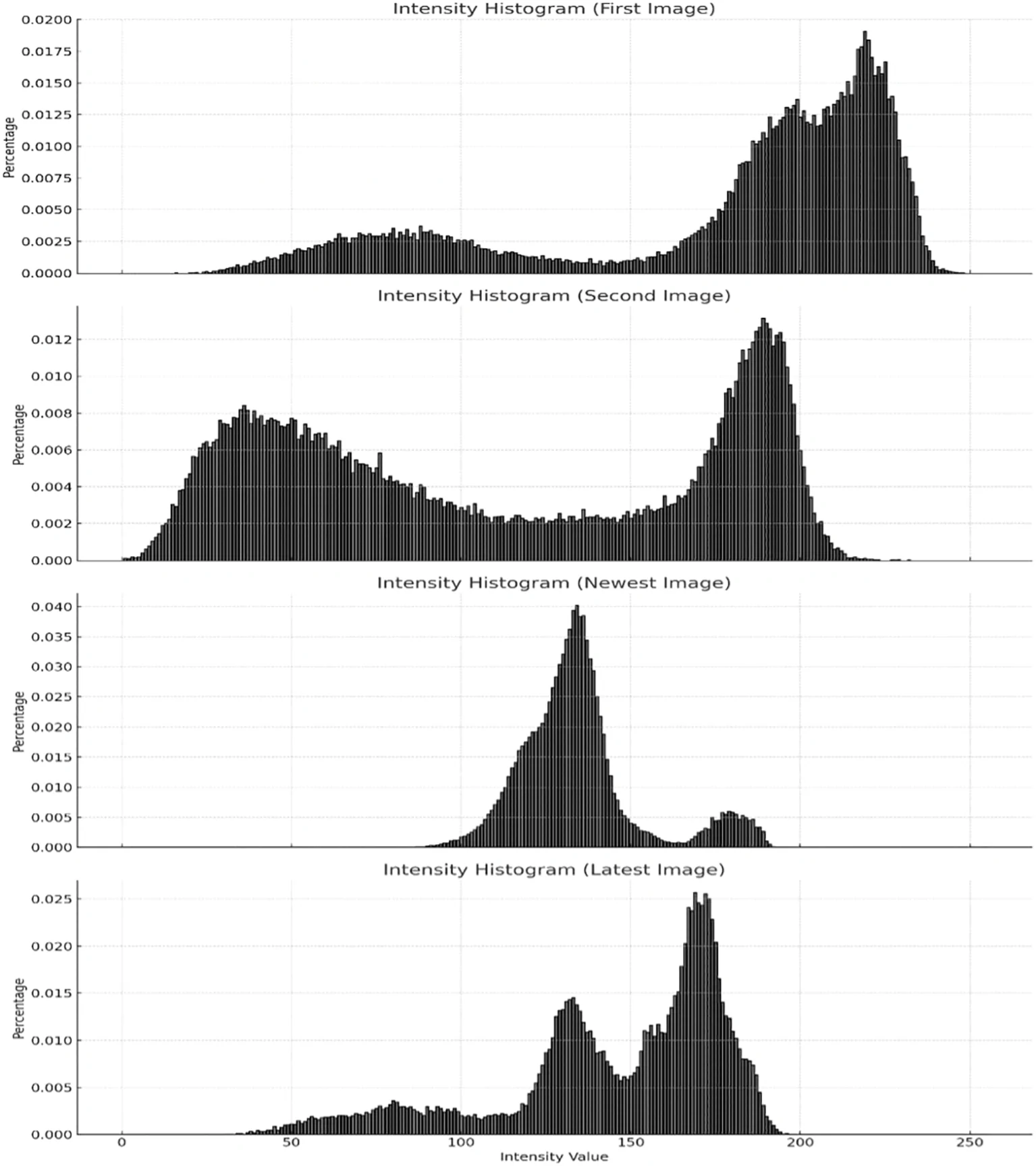
The intensity histograms.

The labeled lines show the assignment to the respective clusters:

- The red one (**Figure 3**) is clearly different from the other images.
- Blue, Green, and Violet (**Figures 4**–**6**) have more similar energy profiles.

Applications:

Clear separation of **Figure 3**:

**Figure 3** (First Image) differs significantly in terms of textures from the other images.

Grouping other images:

**Figures 4**–**6** have more similar texture characteristics, indicating similar energy patterns for the Gabor filter response.

Potential application:

Such analyses can be useful in image classification, diagnosing differences in texture structures (e.g., in medical images), or identifying anomalies.

Further explanation of the analysis:

• Euclidean distances and their meaning (distance matrix):

Euclidean distances calculated between energy profiles (features extracted by Gabor filters) indicate how much the textures vary from image to image:

1. Greatest distance (40,637): Between **Figure 3** (First Image) and **Figure 5** (Newest Image). These images have the most different textures, which may indicate fundamental differences in structure (e.g., different tissues in medical images, altered pathological patterns).
2. Smallest distance (26,808): Between **Figure 4** (Second Image) and **Figure 6** (Latest Image). Means the two images have more similar textures. This may suggest that they are derived from similar materials, tissues, or imaging under similar conditions.

The distances in the matrix allow for an objective assessment of the similarities and differences between images based on the response of the Gabor filters.

Clustering and interpretation of clusters:

As a result of the k-Means analysis, the images were divided into two clusters:

1. Cluster 1:

Includes **Figures 4**–**6**.

These images have more similar energy profiles of the Gabor filters, suggesting that the textures are close to each other.

This may indicate common textural features (e.g., images of different healthy tissues or similar structural characteristics).

2. Cluster 2:

Contains **Figure 3** only.

**Figure 3** has been isolated in a separate cluster, indicating its unique texture characteristics.

Possible explanation: This image may have contained specific patterns, such as an unusual structure (e.g., pathological tissue, different image acquisition method).

3. Importance of the main points in clusters:

**Figure 6** as the hub of Cluster 1:

It is closest to the average values of texture features in cluster 1. It can be considered as a representative of similar images in this cluster.

**Figure 3** is the only one in Cluster 2:

He is unique in his group, indicating that his features do not have close counterparts in the rest of the paintings.

4. Interpretation of the visualization of energy profiles (graph):

The energy profile lines in the graph show how the different Gabor filters (X-axes: Filter Indexes) react to the textural structures in the images:

**Figure 3** (Red):

The energy profile differs significantly from other images (especially in some filter indexes, e.g., 6 and 10), which confirms its difference.

**Figures 4**–**6** (blue, green, purple):

The profiles have more similar shapes, which indicates similarity in the filter responses.

The visualization helps you identify the key filters that differentiate your images the most:

Filters with indices 6, 7, and 10: They show the biggest differences in responses between images.

These filters can be especially important for classifying or diagnosing textures in these images.

Conclusions from the analysis:

1. Image Uniqueness 1:

The energy profile of Image 1 differs so much that it has been assigned to a separate cluster. This can indicate unique texture patterns in the image, such as pathological changes in tissue.

2. Similarities in Cluster 1:

**Figures 4**–**6** have more similar profiles, suggesting common texture features. They can represent healthy tissues, similar materials, or images that are similar in terms of acquisition.

3. Gabor filter value:

Gabor’s filters effectively brought out the texture features that made it possible to distinguish images both visually and numerically.

Filter indexes with the largest differences (e.g., 6, 10) can be particularly useful for further diagnostic analyses.

Results of the in-depth cluster analysis:

1. Average distances in clusters:

Cluster 0 (second image, newest image, latest image):

Average distance inside cluster: 0.0816

The images in this cluster are relatively close to each other, indicating a high similarity in their texture and edge features.

Cluster 1 (first image):

Average distance within cluster: **0.0**

Because this cluster contains only one image, there is no internal differentiation.

2. Average distance between clusters:

Inter-Cluster (Cluster 0 vs. Cluster 1):

Average distance between clusters: 0.2198

It indicates clear differences between the characteristics of the images in these two clusters.

Applications:

1. Similarity within Cluster 0:

Second Image, Newest Image, and Latest Image have similar intensity and edge properties, which may indicate common structures or similar imaging conditions.

2. Image isolation in Cluster 1:

The First Image is significantly different from the others, as evidenced by both the distances between the clusters and previous PCA and clustering analyses.

3. The importance of mean distances:

A small average distance inside clusters (especially Cluster 0) indicates high group cohesion, while a larger distance between clusters highlights the differences between them.

## 6 Gabor filter energy – definition and application

Their energy allows you to quantify the filter’s response to a specific pattern of texture or image structure.

Gabor filter energy is often defined as the average power (or sum of squares) of the filter’s response to an image. The process of calculating energy is as follows:

Calculation steps:


$$G_{real}(x,y)=I(x,y)*g_{real}(x,y)$$

$$G_{imag}(x,y)=I(x,y)*g_{imag}(x,y)$$

Description of the Gabor filter response energy histograms:

X-axis:

Filter Index (Indeks Filtra):

1. On the X-axis are the indexes of the Gabor filters used for image analysis.
2. Each index corresponds to one filter with a specific frequency and orientation.

Y-axis:

Energy:

1. The Y-axis shows the energy value determined for the response of a given Gabor filter.
2. Energy represents the intensity of the filter’s response to textures in an image. Higher values indicate that the filter has better detected a particular texture pattern.

Interpretation of the histograms:

1. Each histogram shows the distribution of response energy for the different Gabor filters applied to the images (first, second, newest, and last).
2. The histogram allows you to understand which filters (frequencies and orientations) are most “active” for a given image, which helps you distinguish textures between different images.

Analysis of the energy histograms of the Gabor filters:

1. First Image:

Maximum energy: 17,501.
Minimum energy: 2,307.
Characteristics: The histogram indicates that certain filters are clearly more “active”, which means that there are dominant texture patterns in the image.

2. Second Image:

Maximum energy: 24,649.
Minimum energy: 2,384.
Characteristics: The response energy for filters is more varied, suggesting a greater variety of textures in the image compared to the first one.

3. Latest Image:

Maximum energy: 28,056.
Minimum energy: 3,897.
Characteristics: The histogram shows the highest energy intensity compared to other images, which may indicate a more complex texture structure.

4. Last Image:

Maximum energy: 24,986.
Minimum energy: 6,474.
Characteristics: The energy is more evenly distributed compared to other images, which can mean more uniform textures.

Applications:

1. Texture Diversity: Each image exhibits different energy distributions of the Gabor filters’ responses, confirming the unique texture characteristics in each image.
2. Maximum Intensity: The latest image has the highest maximum energy, which can indicate clearer or more contrasting texture patterns.
3. Comparability: Differences in histograms can be used to classify images based on their textures.

Description of the interpretation of intensity histograms:

X-axis:

1. Intensity Value:

Displays the pixel intensity values in an image.

The range is typically from 0 to 255, where:

0: Lowest intensity pixels (black).
255: Highest intensity pixels (white).
The intermediate values represent different shades of gray.

Y-axis:

1. Percentage (Procent):

Displays the frequency of pixels of a given intensity in an image as a percentage of the total number of pixels.
This value tells you what percentage of the pixels in the image have a given intensity value.

Meaning of histograms:

The intensity histogram shows the brightness distribution of the pixels in the image:

1. Images with Bright Areas: These have larger values on the right side of the chart.
2. Images with dark areas: These have larger values on the left side of the graph.
3. Wide Distribution: Indicates the presence of a large range of intensities (e.g., a high-contrast image).
4. Narrow Distribution: Indicates a limited range of intensities (e.g., a low-contrast image).

Analysis of intensity histograms:

1. Histograms and visualization:

Histograms show the distribution of pixel intensity in four images. Each of the images has a characteristic intensity distribution profile:

First image: A distribution focused at higher intensities, indicating bright portions of the image.
Second image: A distribution shifted toward low intensities, suggesting darker areas of the image.
Newest image: A more symmetrical distribution, with a peak in the middle intensity range, indicating moderate brightness.
Latest image: A distribution similar to the “Newest Image,” but with more pixels in the higher intensity ranges.

Intensity stats:

Mean intensity:

Indicates average brightness of the image.

First image: 0.778 (highest average, brightest image).
Second image: 0.403 (lowest average, darkest image).
Newest image: 0.588 (moderate brightness).
Latest image: 0.684 (medium brightness image, slightly brighter than “Newest Image”).

Standard deviation of intensity (Std Intensity):

Describes the variation in brightness in an image.

First image: 1.062 (the largest variation in intensity distribution).
Second image: 0.424 (the most homogeneous image).
Newest image: 1.017 (diversity close to “First Image”).
Latest image: 0.980 (moderate variation, closer to “Newest Image”).

2. Key observations:

First image:

The brightest image with the most variability in brightness. It can indicate strongly contrasting light and dark areas.

Second image:

The darkest image with the lowest brightness variation. It probably presents less varied textures.

Newest image:

Image with symmetrical brightness distribution. Indicates a more balanced light intensity in the image.

Latest image:

A moderately bright image with less variation than “first image”. Can represent images with balanced contrast.

## 7 Conclusions

The research presented in this study underscores the critical importance of developing advanced, automated systems for the early detection of skin lesions, specifically malignant melanoma and non-melanoma diseases (1, 3). Given the significant disparity in diagnostic accuracy between experts and non-experts—where experts achieve a 94.2% true negative rate compared to a mere 32.8% for non-experts using the Three-Point Checklist of Dermoscopy (3PCLD)—there is a clear and urgent clinical need for computer-aided diagnostic (CAD) support (3, 4). This work has successfully demonstrated that unsupervised learning methods, combined with rigorous feature extraction of shape and texture, can provide a robust framework for identifying and classifying suspicious lesions (2, 7).

The primary methodological contribution of this paper lies in the integration of unsupervised clusterization with supervised classification to evaluate dermatological asymmetry (7, 9). By extracting asymmetry features as vectors and applying the Dermatological Asymmetry Measure of Shape (DASMShape)—utilizing both exponential and rational function types—the system can numerically quantify the degree of irregularity in a lesion (5, 6, 10). The experimental results across the PH2 dataset revealed that the k-Means (KM) clusterization method provided the most reliable results for identifying lesion centroids and mean values, closely followed by the Canopy method (7, 11). When validated through classification algorithms, the system demonstrated exceptional accuracy; notably, the Random Forest (RF) classifier achieved a 100% success rate across all clusters, while the combination of k-Means and the C4.5 binary tree reached 99.3% (12).

Beyond mere shape analysis, the inclusion of Gabor filter energy profiles has proven to be a decisive factor in distinguishing unique texture patterns (13, 14). The analysis of energy histograms allowed for the quantification of a filter’s response to specific image structures, effectively translating visual textures into a measurable numerical format (15, 21). The study’s deep dive into cluster analysis showed that certain images, such as **Figure 1** in the test set, were isolated into entirely separate clusters due to their unique texture characteristics and high energy intensity (peaking at 17,501) (22–24). This isolation is medically significant, as it suggests that the system can automatically flag lesions with “unusual structures”—such as pathological tissue—that differ fundamentally from common nevi (23, 25).

The use of Euclidean distance matrices further validated the system’s ability to measure similarity objectively (11). For instance, the greatest distance observed between “Images” 1 and 3 (~40,637) confirmed significant structural differences, whereas smaller distances within Cluster 0 indicated high group cohesion and similarity in imaging conditions (11, 15, 26). This mathematical rigor is complemented by intensity histogram analysis, which revealed that mean intensity and standard deviation are vital indicators of a lesion’s character (27, 28). Brightness variability (e.g., Image 1 having the highest average at 0.778 and the largest standard deviation at 1.062) provides an additional layer of diagnostic data regarding contrast and light distribution that simple visual inspection might overlook (16, 28).

Furthermore, the identification of specific Gabor filter indices (specifically (5, 9, 11) as the primary differentiators between clusters suggests that these specific frequencies and orientations are the most “active” in detecting pathological changes (23). This finding allows for the potential optimization of future CAD systems by focusing computational resources on the most relevant filters, thereby reducing the average processing time, which currently ranges from 0.8 to 2.03 seconds per lesion (23, 29).

To sum up, the proposed approach effectively combines shape asymmetry (DASMShape) with texture analysis (Gabor filter energy) and intensity distribution to create a comprehensive diagnostic profile (7, 17). The high classification results—reaching up to 100% in certain configurations—confirm that unsupervised learning can accurately group lesions into diagnostic categories that align with expert assessments (7, 12). This system represents a significant step toward bridging the gap between general practitioners and specialists, providing a tool that prioritizes high sensitivity while maintaining the analytical depth required to reduce false negatives in melanoma screening (3, 7, 30).

Next Steps

Future research will focus on expanding the dataset beyond the PH2 library to include a wider variety of skin tones and rare pathological subtypes to ensure the system’s global applicability e.g. to use the images from Interactive Atlas of Dermoscopy (38) and the International Skin Imaging Collaboration: Melanoma Project (39). We plan to further refine the diagnostic algorithm by integrating DASMHue and DASMStruct measures, allowing for a more nuanced multi-axial analysis of color and internal lesion structures. Finally, the authors intend to implement this automated procedure into a mobile-ready platform to facilitate real-world clinical testing by non-expert medical practitioners.

## Data Availability

The raw data supporting the conclusions of this article will be made available by the authors, without undue reservation.

## References

[1] ACS – American Cancer Society . Available online at: https://www.cancer.org/research/cancer-facts-statistics.html (Accessed December 12, 2025).

[2] European Cancer Information System (ECIS) . Available online at: https://ecis.jrc.ec.europa.eu (Accessed December 12, 2025).

[3] Argenziano G Soyer HP Chimenti S Talamini R Corona R Sera F . Dermoscopy of pigmented skin lesions: results of a consensus meeting via the Internet. J Am Acad Dermatol. (2003) 48:679–93. doi: 10.1067/mjd.2003.281 12734496

[4] Carrera C Marchetti MA Dusza SW Argenziano G Braun RP Halpern AC . Validity and reliability of dermoscopic criteria used to differentiate nevi from melanoma: A web-based international dermoscopy society study. JAMA Dermatol. (2016) 152:798–806. doi: 10.1001/jamadermatol.2016.0624 27074267 PMC5451089

[5] Soyer HP Argenziano G Zalaudek I Corona R Sera F Talamini R . Three-point checklist of dermoscopy. A new screening method for early detection of melanoma. Dermatology. (2004) 208:27–31. doi: 10.1001/jamadermatol.2022.3907 14730233

[6] Argenziano G Fabbrocini G Carli P De Giorgi V Sammarco E Delfino M . Epiluminescence microscopy for the diagnosis of doubtful melanocytic skin lesions. Comparison of the ABCD rule of dermatoscopy and a new 7-point checklist based on pattern analysis. Arch Dermatol. (1998) 134:1563–70. doi: 10.1001/archderm.1997.03890420091011 9875194

[7] Milczarski P Stawska Z Maslanka P . (2017). “ Skin lesions dermatological shape asymmetry measures”, in: Proceedings of the IEEE 9th International Conference on Intelligent Data Acquisition and Advanced Computing Systems: Technology and Applications, IDAACS, 1056–62.

[8] Milczarski P Beczkowski M Borowski N . (2021). “ Blue-white veil classification of dermoscopy images using convolutional neural networks and invariant dataset augmentation”, in: International Conference on Advanced Information Networking and Applications, AINA 2021 (Cham: Springer), 226 LNNS, 421–32.

[9] Was L Milczarski P Stawska Z Wyczechowski M Kot M Wiak S . (2017). “ Analysis of skin diseases using segmentation and color hue in reference to melanocytic lesions”, in: LNCS 10245, Springer, Cham: Springer, 677–89. doi: 10.1007/978-3-319-59063-9_61

[10] Barata C Ruela M Francisco M Mendonça T Marques JS . Two systems for the detection of melanomas in dermoscopy images using texture and color features. IEEE Syst J. (2014) 8:965–79. doi: 10.1109/jsyst.2013.227154025079929

[11] Jain S Jagtap V Pise N . Computer aided melanoma skin cancer detection using image processing. Proc Comput Sci. (2015) 48:735–40. doi: 10.1016/j.procs.2015.04.20938826717

[12] Ra S Suhil M Guruc DS . Segmentation and classification of skin lesions for disease. Proc Comput Sci. (2015) 45:76–85.

[13] Dalila F Zohra A Reda K Hocine C . Segmentation and classification of melanoma and benign. Optik. (2017) 140:749–61. doi: 10.1016/j.ijleo.2017.04.08438826717

[14] Vincent T NgaBenny Y FungaTim K . Determining the asymmetry of skin lesion with fuzzy borders. Comput Biol Medicin. (2005) 35:103–20. doi: 10.1016/j.compbiomed.2003.11.004 15567181

[15] Ramlakhan K Shang Y . (2011). “ A mobile automated skin lesion classification system”, in: ICTAI, 23rd IEEE International Conference on Tools with Artificial Intelligence, 138–41.

[16] Milczarski P Stawska Z Was L Wiak S Kot M . New dermatological asymmetry measure of skin lesions. Int J Neural Networks Advanced Appl. (2027) 4:32–8.Available online at: https://www.naun.org/main/NAUN/neural/2017/a122016-075.pdf.

[17] Milczarski P . (2018). “ Symmetry of hue distribution in the images”, in: Artificial Intelligence and Soft Computing ICAISC'18, 48–61.

[18] Milczarski P . (2025). “ Increasing the classification rates of the trained models using invariant dataset augmentation”, in: Proceedings of the IEEE/CVF International Conference on Computer Vision 2025 (Honolulu, Hawai'i, USA), 1072–81.

[19] Milczarski P Beczkowski M Borowski N . (2021). “ Enhancing dermoscopic features classification in images using invariant dataset augmentation and convolutional neural networks”, in: Neural Information Processing, 28th International Conference, ICONIP 2021, Sanur, Bali, Indonesia, December 8-12, 2021, Proceedings, Part III, Lecture Notes in Computer Science, LNCS (Cham: Springer), 13110, 403–17.

[20] Harrington P . Machine Learning in Action. Manning Publ, (New York: Shelter Island) (2012).

[21] Blum H Nagel RN . Shape description using weighted symmetric axis features. Pattern Recognit. (1978) 10:167–80. doi: 10.1016/0031-3203(78)90025-0

[22] Brady M Asada H . Smoothed local symmetries and their implementation. Int J Robot Res. (1984) 3:36–61. doi: 10.1177/027836498400300302

[23] Cross ADJ Hancock ER . Scale space vector fields for symmetry detection. Image Vision Comput. (1999) 17:337–45. doi: 10.1016/s0262-8856(98)00133-4

[24] Manmatha R Sawhney H . Finding symmetry in intensity images. In: Technical Report. (Amherst MA: University of Massachusetts Amherst) (1997).

[25] Lowe DG . (1999). “ Object recognition from local scale-invariant features”, in: Proc of Int Conf on Computer Vision, 2, 1150–7.

[26] Shen D Ip H Cheung KT Teoh EK . Symmetry detection by generalized complex moments: a close-form solution. IEEE Pattern Anal Mach Intell. (1999) 21:466–76. doi: 10.1109/34.765657

[27] Shen D Ip H Teoh EK . An energy of assymmetry for accurate detection of global reflexion axes. Image Vis Comput. (2001) 19:283–97. doi: 10.1016/s0262-8856(00)00077-9

[28] Zavidovique B Di Gesù V . The S-kernel: A measure of symmetry of objects. Pattern Recognit. (2007) 40:839–52. doi: 10.1016/j.patcog.2006.04.01338826717

[29] Hernández DAG Santiago-Montero R . Border and asymmetry measuring of skin lesion for diagnostic of melanoma using a perimeter ratio. Asian J Comput Sci Inf Technol. (2016) 6:7–13.

[30] Rajpara SM Botello AP Townend J Ormerod AD . Systematic review of dermoscopy and digital dermoscopy/artificial intelligence for the diagnosis of melanoma. Br J Dermatol. (2009) 161:591–604. doi: 10.1111/j.1365-2133.2009.09093.x 19302072

[31] Satheesha TD Sathya-Narayana D Giriprasad M . Review on early detection of melanoma. Proccedings Int J Adv Technol Eng Res. (2012) 2:80–90.

[32] Liu Z Sun J Smith L Smith M Warr R . Distribution quantification on dermoscopy images for computer-assisted diagnosis of cutaneous melanomas. Med Biol Eng Comput. (2012) 50:503–13. doi: 10.1007/s11517-012-0895-7 22438064

[33] Sirakov NM Mete M Chakrader NS . (2011). “ Automatic boundary detection and symmetry calculation in dermoscopy images of skin lesions”, in: 18th IEEE International Conference on Image Processing (Brussels), 1605–8.

[34] Rosendahl C Cameron A McColl I Wilkinson D . Dermatoscopy in routine practice: ‘Chaos and clues’. Aust Fam Physician. (2012) 41:482–7. 22762066

[35] Soyer HP Argenziano G Zalaudek I Hofmann-Wellenhof R . Dermoscopy: The Essentials. 2nd ed. Saunders Ltd. (UK: Elsevier Saunders) (2011).

[36] Stolz W Riemann A Cognetta AB Pillet L Abmayr W Hölzel D . ABCD rule of dermatoscopy: a new practical method for early recognition of Malignant melanoma. Eur J Dermatol. (1994) 4:521–7. doi: 10.3109/9781841847627.012

[37] Mendoncca T Ferreira PM Marques JS Marcal ARS Rozeira J . (2013). “ PH2 – A dermoscopic image database for research and benchmarking”, in: 35th Annual International Conference of the IEEE Engineering in Medicine and Biology Society (EMBC), Osaka, 5437–40. 10.1109/EMBC.2013.6610779 24110966

[38] The International Skin Imaging Collaboration: Melanoma Project . Available online at: http://isdis.net/isic-project (Accessed December 12, 2025).

